# Intubation in a Case of Ectodermal Dysplasia During Surgery: A Case Report

**DOI:** 10.7759/cureus.51504

**Published:** 2024-01-02

**Authors:** Sindhu Geetha, Neeta Verma, Amol Singam

**Affiliations:** 1 Anaesthesiology, Jawaharlal Nehru Medical College, Datta Meghe Institute of Higher Education and Research, Wardha, IND

**Keywords:** multidisciplinary collaboration, craniofacial anomalies, trans mylohyoid/submental intubation, implant rehabilitation, le fort iii advancement, ectodermal dysplasia

## Abstract

Ectodermal dysplasia, a heterogeneous group of rare genetic disorders, is characterized by the aberrant development of ectodermal structures, leading to various clinical anomalies. This case report presents a unique and challenging case of a 33-year-old male with ectodermal dysplasia who underwent Le Fort III advancement and implant rehabilitation surgery to address severe craniofacial and dental deficiencies. This case, characterized by facial dysmorphism, craniofacial anomalies, and the absence of a nasal bone, highlights the complexity of surgical planning required to address these diverse clinical features. The crucial element of this report is the innovative approach to airway management through trans mylohyoid/submental intubation, which successfully navigated the patient's aberrant anatomy. Multidisciplinary collaboration played a pivotal role in achieving a holistic and patient-centered approach. By sharing this case, we aim to provide insights into the nuances of managing complex patients with ectodermal dysplasia, emphasizing the importance of individualized care, innovative techniques, and interdisciplinary teamwork to optimize patient outcomes and contribute to advancing medical knowledge.

## Introduction

Ectodermal dysplasia, a rare genetic disorder characterized by developmental abnormalities of ectodermal structures, presents unique challenges in oral and maxillofacial surgery [1]. These challenges are further compounded when patients with ectodermal dysplasia require complex craniofacial procedures, such as Le Fort III advancement and subsequent implant rehabilitation [2]. This case report presents a compelling clinical scenario involving a 33-year-old male with a long-standing history of ectodermal dysplasia who underwent a Le Fort III advancement procedure and implant rehabilitation for an edentulous jaw. Ectodermal dysplasia, an umbrella term encompassing various genetic subtypes, typically manifests with an array of clinical features that may include congenital anomalies of the hair, teeth, nails, and sweat glands [3]. The severity of these manifestations varies widely among affected individuals, rendering each case a unique and intricate challenge for the medical and surgical team [4].

In this case, the patient's distinctive craniofacial features, facial dysmorphism, the absence of a nasal bone, and bilateral ossicular congenital aplasia posed a complex set of considerations for the surgical team. Furthermore, an edentulous lower jaw and mandibular prognathism necessitated the planning and execution of a multifaceted surgical approach encompassing both craniofacial advancements and implant-based dental rehabilitation. A critical aspect of the case's complexity was the airway management strategy due to the patient's distinct facial anatomy and the anticipated difficulties securing a traditional airway. Employing trans mylohyoid/submental intubation added a layer of intrigue to this already challenging surgical endeavor [5].

This case report offers a valuable contribution to the medical literature, shedding light on the successful management of a patient with ectodermal dysplasia undergoing Le Fort III advancement and implant rehabilitation surgery, specifically focusing on the innovative airway management approach. By sharing this case, we aim to provide insights into the intricacies and nuances of treating such complex patients, ultimately facilitating better care for individuals with similar conditions in the future. Additionally, this report highlights the importance of interdisciplinary collaboration, precise planning, and innovative techniques in oral and maxillofacial surgery, particularly when addressing the diverse needs of patients with rare genetic disorders.

## Case presentation

A 33-year-old male, known to have ectodermal dysplasia since birth, exhibited facial dysmorphism. At 23, he underwent a surgical procedure for nasal deformity under general anesthesia, which involved using a bone graft from the temporalis flap. He was then scheduled for a Le Fort III advancement procedure, followed by implant rehabilitation for an edentulous jaw.

**Figure 1:**
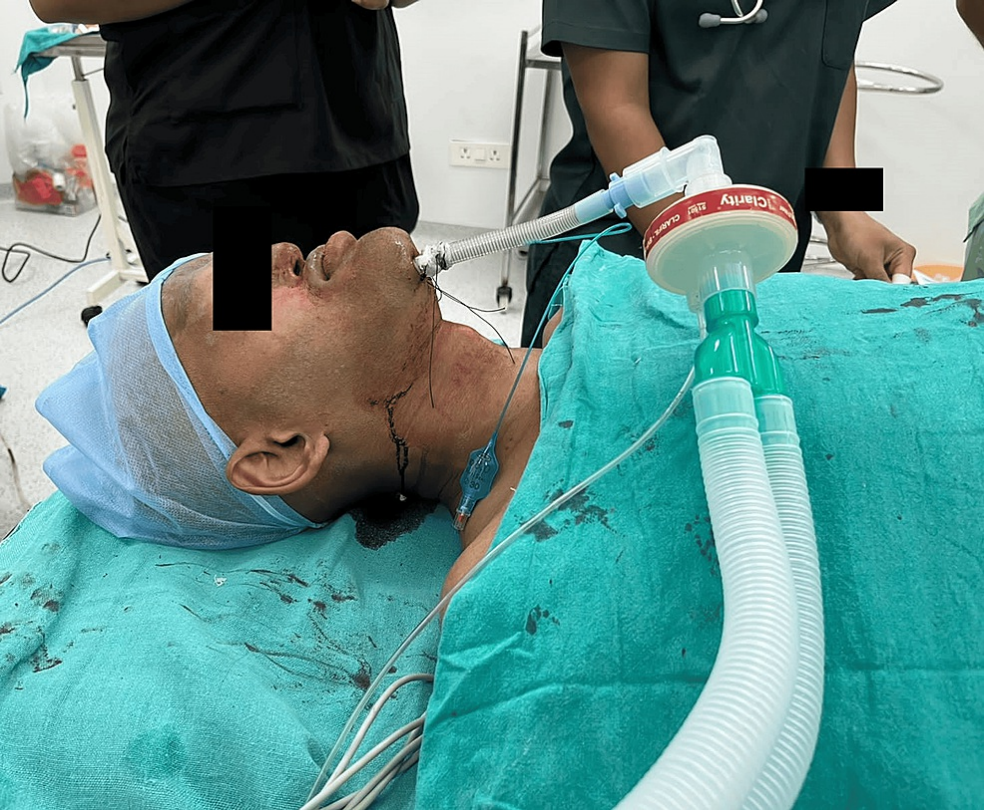
Shows facial asymmetry, an edentulous lower jaw, and mandibular prognathism

During the examination, the patient displayed the following vital signs: he was afebrile, had a pulse rate of 86 beats per minute, and his blood pressure was measured at 110/76 mmHg. The respiratory system examination revealed bilateral equal air entry without any added sounds. Upon assessing the airway, external observations revealed facial asymmetry, a depressed nasal bridge, an edentulous lower jaw, and mandibular prognathism (Figure 1). The 3-3-2 rule was fulfilled, the Mallampati classification was class 2, and there were no signs of obstruction or obesity. Neck mobility was within normal limits.

Laboratory investigations showed hemoglobin levels of 14.4, a total leukocyte count of 4,900, a platelet count of 1.45 lakhs, and a coagulation profile within normal limits. Liver and kidney function tests were also within the normal range. An electrocardiogram showed a normal sinus rhythm, and a chest X-ray displayed no abnormalities. A brain CT scan revealed craniofacial dysmorphism without a nasal bone and bilateral ossicular congenital aplasia. In preparation for the surgery, the patient's consent was verified. All necessary monitors were connected, and two 18-gauge intravenous catheters were secured, one in the left hand and the other in the right. Vital signs were recorded before the induction of anesthesia. Premedications were administered, and the patient was induced with 100 mg of propofol. As the patient was edentulous, gauze pieces covered the cheeks instead of cheek pads. The EC Clamp technique initiated bag and mask ventilation, but a significant leak was observed. A properly sized oropharyngeal airway was inserted, and head tilt and chin lift maneuvers were performed, yet ventilation remained challenging.

**Figure 2:**
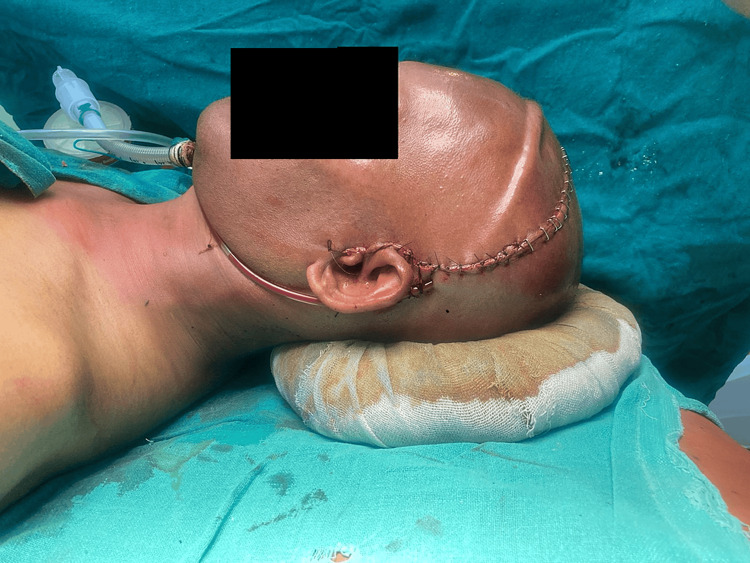
Shows ectodermal dysplasia operated for Le Fort III

Subsequently, the two-hand EC Clamp technique was employed, which allowed for successful ventilation. Vecuronium, 6 mg, was administered intravenously to induce muscle relaxation. The airway was secured through orotracheal intubation, employing an 8.5 mm normal polyvinyl chloride tube and a flexometallic tube via the submental route. Tube placement was confirmed with bilateral auscultation. Intraoperative antibiotics were administered, including a 500 mg loading dose of Tranexamic acid followed by 100 mg hourly to minimize blood loss and a loading dose of 100 mcg of Fentanyl followed by 30 mcg hourly for analgesia throughout the procedure. Vital signs remained stable during the surgery, with an estimated blood loss of approximately 800 ml. The surgical procedure lasted for 14 hours, and the patient was not extubated but was transferred to the ICU with the tube still in place (Figure 2). The following day, the flexometallic tube via the submental route was shifted orally, and elective extubation was performed in the ICU.

## Discussion

Ectodermal dysplasia encompasses a diverse group of genetic disorders characterized by anomalies of ectodermal structures, which can manifest in a wide range of clinical features. Among these features, craniofacial anomalies, including facial dysmorphism, absent nasal bones, and dental abnormalities, are commonly encountered. In our case, the patient presented with a constellation of craniofacial anomalies, emphasizing the importance of an individualized approach to patient care and surgical planning [6]. The Le Fort III advancement procedure is an intricate surgical technique to address midfacial hypoplasia. This case underscores the necessity of considering the impact of ectodermal dysplasia on craniofacial anatomy when planning such procedures. Moreover, the subsequent implant-based dental rehabilitation highlights the importance of comprehensive treatment strategies to address these patients' functional and aesthetic needs [7].

Ectodermal dysplasia often results in facial and oropharyngeal anomalies, challenging airway management during anesthesia. The choice of airway management technique is crucial in ensuring the patient's safety and successful surgery. In this case, traditional bag and mask ventilation was hindered by facial anomalies. The two-hand E-C technique and submental intubation provided an effective solution. This approach allowed for improved access and control of the airway while minimizing the risk of injury or complications associated with other techniques [8,9]. Successful management of complex cases like this one underscores the importance of interdisciplinary collaboration. The involvement of oral and maxillofacial surgeons, anesthesiologists, and other specialists was essential to ensure a comprehensive and patient-centered approach. The cooperation of the surgical and anesthesia teams was particularly crucial in addressing the unique challenges posed by the patient's condition [10].

Sharing this case provides valuable insights for the medical community, as few reports in the literature detail the management of ectodermal dysplasia patients undergoing Le Fort III advancement with implant rehabilitation. It underscores the need for individualized treatment plans and innovative techniques, such as submental intubation, to optimize patient outcomes. Additionally, this case report serves as a reminder of the significance of documenting and sharing unique clinical experiences to advance medical knowledge and patient care [11].

## Conclusions

In conclusion, the case of this 33-year-old male with ectodermal dysplasia who underwent Le Fort III advancement and subsequent implant rehabilitation surgery has provided valuable insights into managing patients with complex craniofacial anomalies. With its wide spectrum of clinical features, Ectodermal dysplasia presents many challenges that demand individualized and multidisciplinary care. The successful execution of the Le Fort III advancement procedure and dental implant rehabilitation addressed the patient's functional and aesthetic needs. It emphasized the significance of precise surgical planning in the context of craniofacial anomalies. Furthermore, the innovative approach of trans mylohyoid/submental intubation for airway management proved effective, serving as a reminder of the importance of adaptability and novel techniques when dealing with intricate cases. Through the amalgamation of surgical expertise and anesthesia coordination, this case report underscores the fundamental role of interdisciplinary collaboration in achieving optimal patient outcomes. Sharing such unique clinical experiences is a contribution to medical knowledge and a testament to the medical community's commitment to advancing patient care, especially for those with rare genetic disorders.
